# Healthcare workers’ compliance and its potential determinants to prevent COVID-19 in public hospitals in Western Ethiopia

**DOI:** 10.1186/s12879-021-06149-w

**Published:** 2021-05-19

**Authors:** Werku Etafa, Gosa Gadisa, Shibiru Jabessa, Tagay Takele

**Affiliations:** 1 Institute of Health Science, Wollega University, Nekemte, Ethiopia; 2 College of Natural and Computational Science, Wollega University, Nekemte, Ethiopia

**Keywords:** Healthcare workers, COVID-19, Prevention, Infection control, Compliance, Barriers, Determinants

## Abstract

**Background:**

Globally, Coronavirus disease-19 has created unprecedented challenges to public health. Healthcare workers (HCWs) are at risk of COVID-19 because of their profession. There are limited studies conducted in Ethiopia among HCWs regarding their compliance with COVID-19 preventive measures. Therefore, this study intended to assess HCWs’ compliance with measures to prevent COVID-19, and its potential determinants in public hospitals in Western Ethiopia.

**Methods:**

A self-administered, multicenter hospital-based cross-sectional survey was proposed to 422 randomly selected HCWs working in seven public hospitals in Western Ethiopia identified as COVID-19 referral centers. Data were entered into Epi Data version 3.1 and analyzed using SPSS version 24. Binary logistic regression was used to identify potential determinants of outcome variables at *p*-value < 0.05.

**Results:**

Out of 422 completed questionnaires, the overall HCWs’ compliance with COVID-19 prevention is 22% (*n* = 404). In multivariate regression analysis, factors such as spending most of caring time at bedside (AOR = 1.94, 95%CI, 1.06–3.55), receiving training on infection prevention/COVID-19 (AOR = 1.86, 95%CI, 1.04–3.33), reading materials on COVID-19 (AOR = 2.04, 95%CI, 1.14–3.63) and having support from hospital management (AOR = 2.09, 95%CI, 1.20–3.64) were found to be significantly associated with COVID-19 preventive measures. Furthermore, inadequate supplies of appropriate personal protective equipment (83.2%), insufficient supportive medications (78.5%), and lack of provision of adequate ventilation (77.7%) were the barriers to COVID-19 prevention most frequently mentioned by participants.

**Conclusion:**

Our findings highlight HCWs’ poor compliance with COVID-19 preventive measures. Providing information and refreshing training to improve the level of healthcare workers’ adherence with COVID-19 prevention is as imperative as increasing staff commitment to supply resources necessary to protect HCWs and to reduce healthcare-associated infections transmission of SARS-COV-2.

**Supplementary Information:**

The online version contains supplementary material available at 10.1186/s12879-021-06149-w.

## Introduction

Coronavirus disease 2019 (COVID-19), is an infectious respiratory disease caused by severe acute respiratory syndrome coronavirus 2 (SARS-CoV-2) [1]. SARS-COV-2 enters into the human cells through angiotensin-converting–enzyme 2 (ACE-2) receptor [2]. The World Health Organization (WHO) declared the COVID-19 outbreak global pandemic on 11 March 2020 [3]. In Ethiopia, the first confirmed COVID-19 case was reported on 13 March 2020 [4].

As of 23 February 2021, globally, over 111 million confirmed cases of COVID-19 and more than 2.4 million deaths have been reported. In the Africa region, over 2.7 million confirmed cases and 70,527 deaths from COVID-19 were notified. Ethiopia reported 152,806 confirmed cases, and 2279 deaths [5]. COVID-19 cases may be understated due to the lack of adequate diagnostic resources to test a larger number of people and a flimsy health care system in low-income countries [6].

The public health measures to prevent and control COVID-19 such as social distancing, self-isolation, and travel restrictions have resulted in a worldwide economic crisis and caused many lost jobs [7]. Healthcare workers (HCWs) are at risk of physical and mental consequences due to COVID-19 patient care [8]. They have a critical role in lowering nosocomial transmission, illness, and death [9, 10] but at the same time they live in the fear of transmitting the virus to their families and community [10].

Despite infection prevention and control (IPC) is the primary role of HCWs, seropositivity for COVID-19 is significantly higher in frontline HCWs working in hospitals, especially among those assigned to COVID-19 wards compared with other frontline HCWs [11, 12]. Considering the risk of COVID-19, protecting HCWs and their families is essential [13].

Previous studies undertaken in Ethiopia showed a poor adherence with COVID-19 prevention among HCWs [14, 15]. Pieces of evidence showed that HCWs’ characteristics such as sex, rural residence, having a chronic illness, and resources related factors such as IPC guidelines, types of healthcare facilities, IPC training, lack of personal protective equipment (PPE), high workload, management support and HCWs’ attitude affect HCWs’ adherence with COVID-19 preventive measures [14–16]. A review of 26 findings [17] identified barriers and facilitators to HCWs’ compliance with IPC guidelines for respiratory infectious diseases, categorized into three domains: organizational factors (safety climate, communication of IPC guidelines and availability of training programs), environmental factors (physical environment and availability of PPE) and individual characteristics such as knowledge, attitude, beliefs, and PPE discomfort.

WHO, Centers for Disease Control and Prevention (CDC), and other governmental organizations have developed guidelines, provided online training sessions and updated information for HCWs. Clinically effective infection prevention and control practices are essential features of patient protection. HCWs are the frontline directly involved in the diagnosis, treatment, and care of COVID-19 patients. This is the best of our knowledge the first study aimed to assess HCWs’ compliance with COVID-19 prevention, its potential determinants and perceived barriers, conducted in public hospitals located in Western Ethiopia.

## Methods

### Study setting, design, and period

A hospital-based cross-sectional survey was conducted from 3 to 28 August 2020 in Wollega zones, Western Ethiopia. The main town of Western Ethiopia is located in the Western part of Oromia National Regional State, 330 km away from Ethiopia’s capital city, Addis Ababa. There are 13 public hospitals in the study area. Among these hospitals there are one specialized (Nekemte Hospital) and one referral hospital (Wollega University Referral Hospital), both located in Nekemte town. In this study, seven public hospitals were included: Nedjo, Mendi, Arjo Jimma, Nekemte specialized, Sire, Shambu, and Guduru. These hospitals were involved in the study because they were identified as COVID-19 referral centers.

### Study population

Study population is formed by all the HCWs working in the above-mentioned seven public hospitals located in Western Ethiopia.

### Sample size determination and sampling procedure

The required sample size was determined by using a single population proportion formula assuming the proportion was 50% (*p* = 0.5) to have a larger sample size. By using a 5% margin of error, we obtained a sample size of 384. Adding a non-response rate of 10%, the final sample became 422.

The total sample was allocated to the selected seven hospitals in proportion to the number of their HCWs. Then, a simple random sampling technique was adopted to draw the study participants after they were proportionally allocated to the selected hospitals, on the base of the staff list obtained from their respective hospitals.

### Data collection tool and procedures

An English language version of a pretested and structured self-administered questionnaire was employed to collect data from the participants. The study tool was adopted from previously published articles and CDC guidelines [15–17].

The self-administered questionnaire used for data collection consists of three parts (Additional file 1). The first one includes demographic and professional characteristics of HCWs (independent variables) such as sex, age, marital status, having child or old family, hospital level, professional type, level of education, work experience, past attendance of training about infection prevention/COVID-19, reading of materials on COVID-19, and whether HCWs’ got support from their hospital management.

The second part of the data collection tool contains 14 items aimed to test HCWs’ compliance with COVID-19 prevention. The reliability of these items had the Cronbach alpha of 0.85. Three experienced research experts, academic health science staff, and hospital staff checked the validity of these items. They were measured on a 3-point Likert scale (1 = seldom, 2 = sometimes, and 3 = always). The final answers were coded ‘1’ for the always answers, and ‘0’ for the sometimes and rarely answers. The score ranges from 0 (the minimum) to 14 (the maximum). HCWs who scored ≥75% were grouped as “good compliance”, and those who scored < 75% were grouped as “poor compliance”. These categories were in line with previously published works [15, 18].

The third part of the questionnaire aimed to identify the perceived barriers to COVID-19 prevention, assessed by a five Likert Scale which assigned ‘1’ for strongly disagree up to ‘5’ for strongly agree.

Two data collection facilitators were recruited for each hospital. Training about COVID-19 prevention and control was given to data facilitators. The packets of questionnaires were distributed to the seven public hospitals by the research team. The study participants were given an oral explanation on the purpose and procedures of the study, the confidentiality of data, a guarantee of voluntary and anonymous participation, and that they could withdraw from the study at any time without fear or prejudice. All HCWs who gave consent were asked to fill in the questionnaire and return it to the data facilitators after compilation. Based on the pretest result from 5% of the estimated sample size at Bako General Hospital located in West Shoa Zone, modifications have been made to avoid ambiguity in the questionnaire.

### Data processing and analysis

Data were entered into EPI data version 3.1 and analyzed by Statistical Package for Social Sciences (SPSS) version 20.0. The descriptive statistics were summarized using tables, figures, and texts, while continuous variables were presented by mean and the standard deviation. To assess the association between independent variables and outcome, we employed binary logistic regression. Odds ratio (OR) with 95% CI was used to determine the strength of association, and *p*-value *<* 0.05 for statistical significance of compliance with COVID-19 prevention.

## Results

### Demographic and professional characteristics

The randomly selected HCWs (422) are representative of the population of HCWs working in the seven hospitals involved in the study (912). In this study, 404 HCWs completed the questionnaires with a response rate of 95.3%. Nearly half of them (48.5%) were found in the age range of 20–29 years with the mean 31.2(±6.24SD). About 45% of participants worked in primary hospitals while most of them (68.1%) were male. Similarly, about 63 % (63.4%) of the participants received training and more than half (58.2%) read materials on COVID-19. Fifty-five percent of them appreciated their hospital management support role in infection prevention and control (Table 1).

**Table 1:** Demographic and professional characteristics of healthcare workers in public hospitals in Wollega Zones, 2020 (*N* = 404)

Variables	Category	Frequency (N)	Percentages (%)
Sex	Male	275	68.1
	Female	129	31.9
Age (years) (mean ± SD, 31.2 ± 6.24) (range: 21–58)	20–29	196	48.5
	30–39	163	40.3
	≥40	45	11.1
Marital status	Married	289	71.5
	Single	113	28.0
	Others^a^	2	0.5
Having child/old	Yes	280	69.3
	No	124	30.7
Level of hospital	Primary	180	44.6
	General	174	43.1
	Specialized	50	12.4
Professional occupation	Medical doctor	50	12.4
	Nurse	171	42.3
	Midwife	72	17.8
	Pharmacist	45	11.1
	Medical laboratory technician	40	9.9
	Others^b^	26	6.4
Professional qualification level	MD (GP and above)	50	12.4
	Masters degree	35	8.6
	Degree	246	60.9
	Diploma	73	18.1
Received training on infection prevention/COVID-19	Yes	256	63.4
	No	148	36.6
Read materials on COVID-19	Yes	235	58.2
	No	169	41.8
Hospital management support	Yes	222	55.0
	No	182	45.0

Others^a^: widowed, divorced and cohabitating; others^b^: environmental health, occupational health and anesthesia

### Healthcare workers’ compliance with COVID-19 preventive measures

**Fig. 1:**
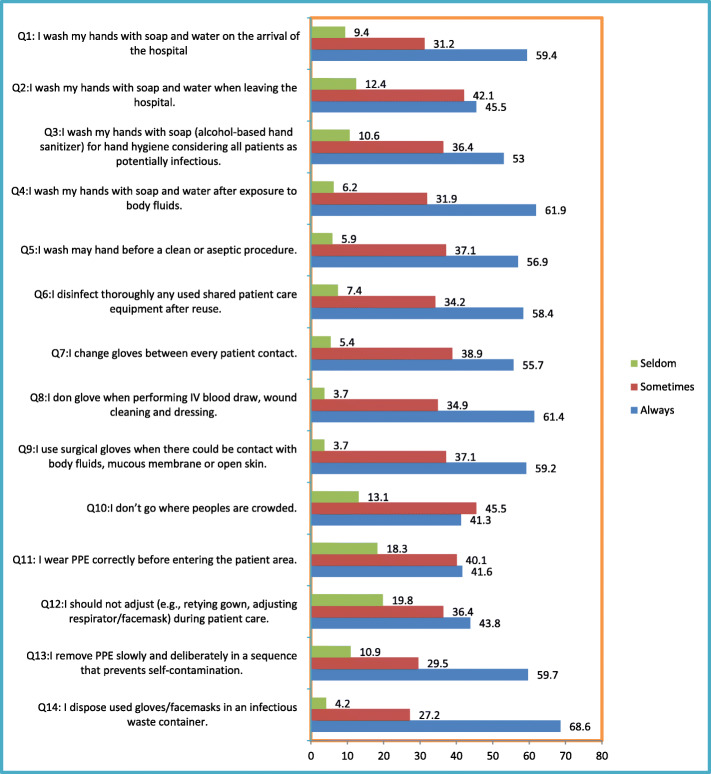
Healthcare workers’ compliance with COVID-19 preventive measures in public hospitals in Western Ethiopia, 2020

Less than one fourth (22%) of HCWs included in the study have adhered to COVID-19 preventive measures. Most of them (*n* = 277, 68.6%) showed better compliance with disposing used gloves/facemasks in an infectious waste container, donning gloves when performing intravenous blood draw, wound clean, and dressing (*n* = 250, 61.9%), and washing hands with soap and water after exposure to body fluids (n = 250, 61.9%). Meanwhile, more than half of them paid less attention to other COVID-19 preventive measures so that we found the following results: inability to avoid going where peoples are crowded 58.6% (*n* = 237), failing in wearing t PPE correctly before entering the patient area 58.4% (*n* = 236), and adjusting (e.g., retying gown, adjusting respirator/face mask) during patient care 56.2% (*n* = 227) (Fig. 1).

### Potential determinants of HCWs’ compliance with COVID-19 preventive measures

In multivariate logistic regression, spending most of caring time at bedside, receiving training about infection prevention/COVID-19, reading materials about COVID-19, and having support from hospital management were independent variables associated with HCWs’ compliance COVID-19 preventive measures.

The odds of having good compliance with COVID-19 preventive measures were about twice as likely among HCWs who spent most of their caring time at bedside, received training on infection prevention/COVID-19 reading materials on COVID-19 had support from their hospital management (Table 2).

**Table 2:** Potential determinants of healthcare workers’ compliance with COVID-19 preventive measures

Variables	Good Compliance	Poor Compliance	COR, 95%CI	AOR, 95% CI	***P***-value
Spending most of caring time at bedside					
Yes	72 (24.6)	221 (75.4)	1.80 (1.008–3.22)	1.94 [1.06–3.55]*	0.03
No	17 (15.3)	94 (84.7)	1.00	1.00	
Receiving training on infection prevention/COVID-19					
Yes	70 (27.3)	186 (72.7)	2.55 (1.46–4.44)	1.86 [1.04–3.33]*	0.03
No	19 (12.8)	129 (87.2)	1.00	1.00	
Reading materials on COVID-19					
Yes	68 (28.9)	167 (71.1)	2.87 (1.67–4.9)	2.04 [1.14–3.63]*	0.01
No	21 (12.4)	148 (87.6)	1.00		
Having support from hospital management					
Yes	65 (29.3)	157 (70.7)	2.72 (1.62–4.57)	2.09 [1.20–3.64]*	0.009
No	24 (13.2)	158 (86.8)	1.00		

*: indicates significant variables at *p*-value < 0.05 in multivariate logistic regression

### Perceived barriers to implement COVID-19 preventive measures

A descriptive analysis identified the most common barriers to implementing COVID-19 preventive measures. The most frequently barriers perceived by HCWs were inadequate supplies of appropriate PPE (including required standards) (*n* = 336, 83.2%), insufficient supportive medications (*n* = 317, 78.5%), lack of provision of adequate ventilation (*n* = 314, 77.7%), lack of sufficient room/space to isolate patients (*n* = 306, 75.7%) and uncooperative community (*n* = 302, 74.7%) (Table 3).

**Table 3:** Perceived barriers to COVID-19 preventive measures in public hospitals in Wollega Zones, 2020 (*N* = 404)

Variables	SDA, n(%)	DA, n(%)	US, n(%)	A, n(%)	SA, n(%)
Inadequate supplies of appropriate PPE (including required standard)	15(3.7)	40(9.9)	13(3.2)	135(33.4)	201(49.8)
Lack of provision of adequate ventilation	27(6.7)	41(10.1)	22(5.4)	154(38.1)	160(39.6)
Inadequate supportive medications	22(5.4)	45(11.1)	20(5.0)	180(44.6)	137(33.9)
Poor access to hand washing facilities and surface decontamination supplies	35(8.7)	58(14.4)	24(5.9)	160(39.6)	127(31.4)
Guidelines (absence, unclear, impractical or not constant)	19(4.7)	61(15.1)	40(9.9)	159(39.4)	125(30.9)
Staff shortage which increases workload	26(6.4)	48(11.9)	36(8.9)	132(32.7)	162(40.1)
Instability/conflicts in the area	31(7.7)	63(15.6)	63(15.6)	125(30.9)	122(30.2)
Lack of updated information	27(6.7)	69(17.1)	42(10.4)	147(36.4)	119(29.5)
Lack of adequate training	27(6.7)	54(13.4)	22(5.4)	129(31.9)	172(42.6)
Lack of sufficient room/space to isolate patients	37(9.2)	32(7.9)	29(7.2)	142(35.1)	164(40.6)
Communication gap with higher health officials	34(8.4)	78(19.3)	58(14.4)	125(30.9)	109(27.0)
Uncooperative community	22(5.4)	44(10.9)	36(8.9)	173(42.8)	129(31.9)
Limited knowledge of healthcare workers	27(6.7)	108(26.7)	62(15.3)	125(30.9)	82(20.3)
Healthcare workers’ belief/fear of infecting themselves	23(5.7)	53(13.1)	40(9.9)	158(39.1)	130(32.2)

*SDA* Strongly disagree, *DA* Disagree, *US* Unsure, *A* Agree, *SA* Strongly agree

## Discussion

COVID-19 is an emerging pandemic infectious disease of global public health concern. It is the most current topic of discussion across every individual life, especially among HCWs and patients. HCWs are at higher risk for infection by SARS-CoV-2 than the general population.

The present cross-sectional study was intended to assess HCWs’ compliance with COVID-19 prevention practices in public hospitals in Western Ethiopia and its potential determinants. Overall reported compliance observed in our study was poor (22%). This result is lower than those reported in the Central Gondar zone (38.7%) and Amhara region (62%), Ethiopia, and Uganda (74%), China (89%), and Pakistan (73%) [13–15, 19, 20]. This difference might be due to political instability in the area that entails the scarcity of resources for COVID-19 prevention. The disparity in study methods and economic level compared with other countries could also be another possible reason.

In our study, spending most of the caring time with inpatients intensifies HCWs’ engagement in self-prevention from infection caused by SARS-CoV-2. However, there were no similar studies that examined this type of association. This result suggests that fear infection strengthens HCWs’ ability to protect from exposure risk to SARS-CoV-2. Thus, since HCWs’ self-prevention practice is a model for their patients and attendants in preventing healthcare-associated infections, it should be encouraged. Likewise, HCWs who received training on COVID-19 preventive measures showed good compliance with COVID-19 prevention than HCWs who did not. This is consistent with the study conducted in the Amhara region [14]. Since COVID-19 is a newly emerged disease with little information about it, appropriate training can improve HCWs’ knowledge and skills on this pandemic disease. Staff should be committed to the implementation of infection prevention and control strategies.

In addition, reading resources on COVID-19 and obtaining support from the hospital management are other factors requiring emphasis to improve HCWs’ compliance with COVID-19 prevention. Through reading materials on COVID-19, HCWs can enhance their knowledge and ability to protect themselves and reduce healthcare transmission. Therefore, hospital administrations should HCWs’ access about COVID-19 like access to internets at the workplace. It is in harmony with the study finding from Gondar University, Ethiopia, on standard precaution aimed at infection prevention [19]. Hospital management should be strongly involved and required to support HCWs by supplying essential equipment to prevent COVID-19 and providing psychosocial support to HCWs.

Furthermore, this study identified the most common barriers perceived by HCWs to implement COVID-19 preventive measures. Inadequate supplies of appropriate PPE (including required standards) is the most frequently (83.2%) cited barrier to adopt COVID-19 preventive measures in public hospitals in Wollega zones. This issue is also of concern to HCWs in different countries [11, 13]. Lack of PPE can significantly hamper HCWs’ adherence with infection prevention and control despite their knowledge. From our experience, inadequate supply of PPE in developing countries such as Ethiopia is unsurprising. This could be due to an insufficient hospital budget unable to provide high-quality PPE in appropriate quantities. Inadequacy of PPE supply could prompt HCWs to use it for extended periods or recycle PPE as a desperate means to keep it [21]. Therefore, hospital management and administration are expected to increase during COVID-19 pandemic period than ever. For example, the Spanish government has faced this pandemic by allocating new funds, purchasing and introducing price controls, and buying relevant equipment appropriate to COVID-19 prevention [22].

In this study, HCWs reported an insufficient supply of supportive medications useful in controlling COVID-19 worsening. The evidence showed that although various drugs are being studied around the globe, in the USA the Food and Drug Administration approved an antiviral agent (Remdesivir) for COVID-19 treatment [23]. For low-income countries, it is challenging to invest in such drugs as its requirement is huge.

Lack of adequate ventilation is another barrier reported by more than three-quarters of the HCWs. This is an issue also mentioned by HCWs in a different country [11, 13]. Since poorly ventilated wards/units and waiting rooms put individuals at risk of acquiring infection by SARS-CoV-2, public hospitals should increase ventilation equipment and decrease the number of visitors to avoid overcrowding.

Lack of appropriate physical space (insufficient room) to isolate patients is also a common issue highlighted by a large proportion of HCWs in the study area. This can be impacted by high patient turnover at the time of the crisis. There is evidence that an adequate isolation room is the best approach to minimize cross-contamination [24]. Working on the extension of public hospital infrastructure to maintain physical space for COVID-19 prevention and control is suggested [25].

HCWs included in this study have cited uncooperative community as another obstacle to COVID-19 prevention. Considering that COVID-19 preventive measures require teamwork, community involvement is of significant concern. If communities are not informed and educated, they might consider visiting their hospitalized relatives and friends as their usual habits. Therefore, providing appropriate information and instruction to ensure the necessary precautions practiced by the community is fundamental.

## Conclusions

In our study, we found poor HCWs’ compliance with COVID-19 preventive measures. Potential determinants of good compliance with COVID-19 preventive measures were HCWs who are frontline, receiving training about infection prevention/COVID-19, reading materials on COVID-19, and having support from hospital management. This study identified the most frequent barriers perceived by HCWs’ to prevent COVID-19: inadequate supplies of appropriate PPE (including required standards), insufficient supportive medications, lack of adequate ventilation, sufficient room/space to isolate patients, and uncooperative community.

### Limitations of the study

The cross-sectional study design allows determining associations but not causal relationships in the analysis of potential determinants of HCWs’ compliance with COVID-19 preventive measures. Also, a self-administered questionnaire has some limitations like social desirability bias (respondent may respond in a socially acceptable way). Moreover, insufficient data are available for comparing our findings with others.

## Supplementary Information

**Additional file 1.**

## Data Availability

The datasets used and/or analysed during the current study are available from the corresponding author on reasonable request.

## Abbreviations

ACE-2: Angiotensin-converting–enzyme 2
CDC: Centers for Disease Control and Prevention
COVID-19: Coronavirus disease 2019
HCWs: Healthcare workers
IPC: Infection prevention and control
IP: Infection prevention
IV: Intravenous
PPE: Personal protective equipment
SARS-CoV-2: Severe acute respiratory syndrome coronavirus 2
SPSS: Statistical package for social sciences
WHO: World Health Organization
