# Supplementary material for: Healthcare workers’ compliance and its potential determinants to prevent COVID-19 in public hospitals in Western Ethiopia
Source: BMC Infect Dis. 2021 May 19;21:454. doi: 10.1186/s12879-021-06149-w (PMC8132019; doi:10.1186/s12879-021-06149-w)
Supplement: Supplementary file 1 — Additional file 1. [file 12879_2021_6149_MOESM1_ESM.docx]

**WOLLEGA UNIVERSITY INISTITUTE OF HEALTH SCIENCE**

**Questionnaire prepared to assess “Healthcare workers’ compliance and its potential determinants to prevent COVID-19 in public hospitals in Western Ethiopia”.**

**Instruction I: It focuses on demographic and professional characteristics of healthcare workers. Please encircle to the number or your appropriate answer in the space provided.**

Name of your hospital: ____________________________

| Variables | **Options** | **Response** |
| --- | --- | --- |
| 1. What is your sex? | 1. Male 2. Female |  |
| 1. How old are you? | I am___________ years old |  |
| 1. What is your marital status | 1. Married 2. Single 3. Widowed/divorced |  |
| 1. Do you have an old people/child at home? | 1. Yes 2. No |  |
| 1. What is the level of your hospital? | 1. Primary 2. General 3. Specialized/Referral |  |
| 1. What is your professional occupation? | 1. Medical Doctor 2. Nurse 3. Midwife 4. Pharmacist 5. Medical laboratory 6. Others______________ |  |
| 1. Which is your professional qualification level? | 1. MD/MD+ 2. MSc 3. BSc 4. Diploma and below |  |
| 1. Have you ever received training on infection prevention/COVID 19? | 1. Yes 2. No |  |
| 1. Did you read informational materials (e.g., articles, brochures, guidelines) on COVID-19? | 1. Yes 2. No |  |
| 1. Does your hospital management support safety environment of your hospital practically? | 1. Yes 2. No |  |

**Instruction III: Answer the following questions by marking (✓) in corresponding column according to frequency of your performance/practice/ to assess compliance level of healthcare workers in public hospitals in Wollega Zones, 2020. The options for responses are always, sometimes and never.**

| **Compliance testing activities** | **Always** | **Some**  **times** | **Never** |
| --- | --- | --- | --- |
| 1. I wash my hands with soap and water on the arrival of the hospital. |  |  |  |
| 1. I wash my hands with soap and water when leaving the hospital. |  |  |  |
| 1. I wash my hands with soap and water or use alcohol-based hand sanitizer considering all patients as potentially infectious. |  |  |  |
| 1. I wash my hands with soap and water after exposure to body fluids. |  |  |  |
| 1. I wash my hands before a clean or aseptic procedure. |  |  |  |
| 1. I disinfect thoroughly any used shared patient care equipment after reuse. |  |  |  |
| 1. I change gloves between every patient contact. |  |  |  |
| 1. I don gloves when performing intravenous (IV) blood draw, wound cleansing and dressing. |  |  |  |
| 1. I use gloves when there could be contact with body fluids, mucus membrane or open skin. |  |  |  |
| 1. I don’t go where peoples are crowded. |  |  |  |
| 1. I wear personal protective equipment correctly before entering the patient area. |  |  |  |
| 1. I should not adjust (e.g., retying gown, adjusting respirator/facemask) during patient care. |  |  |  |
| 1. I remove personal protective equipment slowly and deliberately in a sequence that prevents self-contamination. |  |  |  |
| 1. I dispose used gloves/facemasks in infectious waste container. |  |  |  |

**Instruction III: Following COVID-19 preventive measures, please choose by ticking (✓)** **the challenge/barrier to prevent COVID-19 in your hospital in the column from strongly agree (SA) to strongly disagree (SDA) as shown below. NB: SA= Strongly agree, A= Agree, US= Unsure, DA=Disagree & SDA= strongly disagree.**

| **Challenges/barriers** | **SA** | **A** | **US** | **DA** | **SDA** |
| --- | --- | --- | --- | --- | --- |
| 1. Inadequate supplies of appropriate PPE (including required standard) |  |  |  |  |  |
| 1. Lack of provision of adequate ventilation |  |  |  |  |  |
| 1. Inadequate supportive medications |  |  |  |  |  |
| 1. Poor access to hand washing facilities and surface decontamination supplies |  |  |  |  |  |
| 1. Guidelines (absence, unclear, impractical or not constant) |  |  |  |  |  |
| 1. Staff shortage which increases workload |  |  |  |  |  |
| 1. Instability/conflicts in the area |  |  |  |  |  |
| 1. Lack of updated information |  |  |  |  |  |
| 1. Lack of adequate training |  |  |  |  |  |
| 1. Lack of sufficient room/space to isolate patients |  |  |  |  |  |
| 1. Communication gap with higher health officials (like Ministry of Health or Regional Health officials) |  |  |  |  |  |
| 1. Uncooperative community (to minimize overcrowding, visitors and fast-tracking infected patients) |  |  |  |  |  |
| 1. Limited knowledge of healthcare workers |  |  |  |  |  |
| 1. Healthcare workers’ belief/fear of infecting themselves |  |  |  |  |  |

**Thank you!**
